# Alternative C3 Complement System: Lipids and Atherosclerosis

**DOI:** 10.3390/ijms22105122

**Published:** 2021-05-12

**Authors:** Maisa Garcia-Arguinzonis, Elisa Diaz-Riera, Esther Peña, Rafael Escate, Oriol Juan-Babot, Pedro Mata, Lina Badimon, Teresa Padro

**Affiliations:** 1 Cardiovascular Program-ICCC, Research Institute-Hospital Santa Creu i Sant Pau, IIB-Sant Pau, 08025 Barcelona, Spain; mgarciaar@santpau.cat (M.G.-A.); ediazr@santpau.cat (E.D.-R.); epena@santpau.cat (E.P.); rescate@santpau.cat (R.E.); ojuan@santpau.cat (O.J.-B.); lbadimon@santpau.cat (L.B.); 2 Centro de Investigación Biomédica en Red Cardiovascular (CIBERCV), Instituto de Salud Carlos III, 28029 Madrid, Spain; 3 Fundación Hipercolesterolemia Familiar, 28010 Madrid, Spain; pmata@colesterolfamiliar.org; 4 Cardiovascular Research Chair, UAB, 08025 Barcelona, Spain

**Keywords:** atherosclerosis, cardiovascular disease, complement system, proteomics, mass spectrometry

## Abstract

Familial hypercholesterolemia (FH) is increasingly associated with inflammation, a phenotype that persists despite treatment with lipid lowering therapies. The alternative C3 complement system (C3), as a key inflammatory mediator, seems to be involved in the atherosclerotic process; however, the relationship between C3 and lipids during plaque progression remains unknown. The aim of the study was to investigate by a systems biology approach the role of C3 in relation to lipoprotein levels during atherosclerosis (AT) progression and to gain a better understanding on the effects of C3 products on the phenotype and function of human lipid-loaded vascular smooth muscle cells (VSMCs). By mass spectrometry and differential proteomics, we found the extracellular matrix (ECM) of human aortas to be enriched in active components of the C3 complement system, with a significantly different proteomic signature in AT segments. Thus, C3 products were more abundant in AT-ECM than in macroscopically normal segments. Furthermore, circulating C3 levels were significantly elevated in FH patients with subclinical coronary AT, evidenced by computed tomographic angiography. However, no correlation was identified between circulating C3 levels and the increase in plaque burden, indicating a local regulation of the C3 in AT arteries. In cell culture studies of human VSMCs, we evidenced the expression of C3, C3aR (anaphylatoxin receptor) and the integrin α_M_β_2_ receptor for C3b/iC3b (RT-PCR and Western blot). C3mRNA was up-regulated in lipid-loaded human VSMCs, and C3 protein significantly increased in cell culture supernatants, indicating that the C3 products in the AT-ECM have a local vessel-wall niche. Interestingly, C3a and iC3b (C3 active fragments) have functional effects on VSMCs, significantly reversing the inhibition of VSMC migration induced by aggregated LDL and stimulating cell spreading, organization of F-actin stress fibers and attachment during the adhesion of lipid-loaded human VSMCs. This study, by using a systems biology approach, identified molecular processes involving the C3 complement system in vascular remodeling and in the progression of advanced human atherosclerotic lesions.

## 1. Introduction

Familial hypercholesterolemia (FH), an autosomal-dominant disorder mainly caused by the loss-of-function mutations in the low-density lipoprotein (LDL) receptor, is associated with an increased risk of atherosclerosis and ultimately premature cardiovascular event presentation, resulting in lifelong exposure to high-LDL cholesterol levels [1,2,3,4,5]. Increasing evidence suggests that FH patients recurrently present an inflammatory phenotype that is maintained despite treatment with lipid lowering therapies according to guidelines [6,7,8,9]. We and others have demonstrated that adult FH patients have higher levels of extracellular microvesicles originating from inflammatory cells in plasma [6] as well as circulating mononuclear cells and monocyte-derived macrophages with inflammatory phenotypes [7,10].

The complement system is an important component of the innate immunity and plays a key role in the regulation of inflammation. Particularly relevant is the activation of the alternative C3 system, in which the different pathways of the complement system converge, leading to the formation of active C3 proteolytic products, C5 convertases and eventually the activation of the terminal complement proteins C5 to C9 and the formation of the membrane attack complex (MAC) [11,12]. The C3 system is tightly regulated by a cascade of components, including activators (Factor B), inhibitors (Factor H) and cell surface proteins acting as receptors (CR1, C3aR and α_M_β_2_ integrin).

Atherosclerosis is widely recognized as a lipid-induced chronic inflammatory disease of the arterial wall with the activation of resident cells and recruitment of circulating leukocytes [13]. The complement system has been repeatedly associated with vascular remodeling [14] and atherosclerosis [15]. Results from experimental animal models and human samples suggest that complement activation may exert dual atheroprotective and proatherogenic effects mainly associated with the initial and terminal stages of the complement cascade, respectively [16,17]. However, the impact of the C3 complement system in atherogenesis is not fully understood.

C3 complement products and their cell receptors have been detected by immunohistochemistry in areas with atherosclerotic lesions of different severity in human arteries [18,19], which has led to the hypothesis that local activation of the alternative-complement system is involved in atherosclerotic plaque progression and complication [15]. In contrast, prior studies in mice models of atherosclerosis (*Ldlr*-/- or *ApoE*-/- *Ldlr*-/- background) and knock-out C3 expression (*C3*-/-) evidenced that atherosclerotic lesions developed in the absence of C3 have a lower content of vascular smooth muscle cells (VSMCs) and collagen, hallmark of vulnerable plaques, and are of a larger size than those plaques developed in animals with a sufficient content of C3 [20,21]. A potential effect of C3 on the proliferation of VSMCs during atherogenesis was suggested by a recent study, in *ApoE*-/- mice fed a high-fat Western diet, in which dedifferentiated clonally expanding vascular SMC showed an up-regulated C3 expression [22] and also by prior results linking C3a with the increasing proliferation of mouse VSMCs [23].

Results from two recent studies evidenced a noticeable increase in arterial wall inflammation, assessed by fluorodeoxyglucose positron emission tomography imaging, in FH patients with high LDL levels with healthy controls [24,25]. The up-regulation of components of the complement cascade, including C3-derived products, have been reported in two studies in FH patients with no clinical evidence of coronary artery disease [23,26]. To date, however, we do not know whether circulating C3 levels relate to the intensity and profile of atherosclerotic plaque burden. Moreover, little is known regarding the interplay among LDL, C3 complement products and VSMCs, although VSMCs are the key cellular components in the development and complication of atherosclerotic lesions.

Therefore, the present study was conducted to investigate the relationship between circulating C3 complement, lipids and atherosclerotic plaque burden in FH patients with subclinical atherosclerosis. In addition, using a mass spectrometry-based proteomic approach, combined with transcriptomic analysis and in vitro functional assays, we analyzed the pattern of the C3 complement components in the extracellular matrix (ECM) of human atherosclerotic plaques and investigated C3 complement expression and effects on migration kinetics of lipid-loaded VSMCs.

## 2. Results

### 2.1. Characteristics of the FH Patient Population

FH patients from the SAFEHEART cohort were included (*N* = 49; 31 men and 18 women). The mean age was 44.7 ± 10.5 years (men: 45.4 ± 11.4 years; women 43.7 ± 9.2 years). The baseline demographic and clinical characteristics of the studied FH-population are shown in Table 1. All subjects were on lipid-lowering treatment (LLT) and treated with statins as per the guidelines for > 1 year (mean treated years before inclusion in the study were 14.9 ± 6.7 years). The mean LDL-cholesterol (LDL-C) in the FH group was 136.3 ± 36.0 mg/dL.

None of the FH patients had a clinical history of cardiovascular disease (CVD). Less than 5% of patients with FH presented hypertension or Type-2 diabetes. Eleven FH patients (22%) were active tobacco smokers. The mean value for 5- and 10-year CVD risk in the FH patients, according the SAFEHEART-risk score (SAFEHEART-RS), was 1.00 ± 0.76 % and 2.13 ± 1.6 %, respectively.

All FH subjects included in the study presented subclinical atherosclerosis, assessed by computed tomographic angiography (CTA) and quantified by SAPC software [27]. The mean value for the total plaque burden was 23.5 ± 6.3% and, specifically, the median calcified plaque burden was 2.2 ± 2.5%, and that of non-calcified-plaque burden was 21.3 ± 5.3%. FH patients with plaque burden above the median values (high plaque burden) had a significantly higher estimated cardiovascular risk based on SAFEHEART-RS, both at 5 (0.77 ± 0.76% vs. 1.24 ± 0.15%; *p* = 0.03) and 10 years (1.65 ± 1.59% vs. 2.60 ± 0.15%; *p* = 0.03).

### 2.2. C3 Complement in Patients with Hypercholesterolemia and Subclinical Atherosclerosis

**Figure 1:**
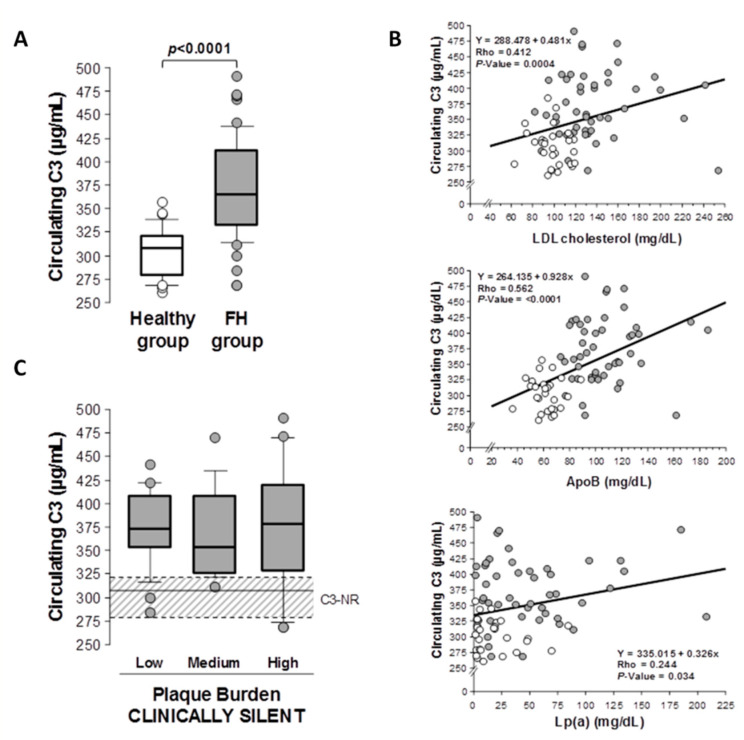
Circulating C3 complement in patients with hypercholesterolemia and subclinical atherosclerosis. (**A**) Plasma circulating C3 complement (µg/mL) in subjects with genetic diagnosis of FH and subclinical atherosclerosis (*n* = 49) compared to levels in a young healthy population (*n* = 28). (**B**) Correlation between plasma C3 levels and LDL-C, ApoB and Lp(a) levels in the study population (FH patients with subclinical atherosclerosis and healthy population). (**C**) Circulating C3 complement by plaque burden tertiles in FH patients. Dashed box (C3-NR) indicates the normal range of C3 levels in a healthy population (*n* = 28). Results are shown as median ± SE. *p* < 0.05 was considered statistically significant (Mann–Whitney and Kruskal–Wallis tests).

Circulating levels of C3 complement were significantly elevated (*p* < 0.001) in subjects with a genetic diagnosis of FH and subclinical coronary atherosclerosis, when compared to the plasma levels of C3 in young healthy subjects at low atherosclerotic risk (subjects without CV risk factors and age between 18 and 35 years) (Figure 1A). Plasma C3 complement levels were significantly correlated with LDL-C levels (Spearman correlation: Rho value = 0.412, *p* < 0.001), ApoB levels (Spearman correlation: Rho = 0.562, *p* < 0.001) and Lp(a) (Spearman correlation: Rho = 0.244, *p* = 0.034) when the whole study population was considered (healthy subjects and FH patients) (Figure 1B). No significant correlation was found with other lipid variables, including triglycerides (Spearman correlation: =0.021, *p* = 0.856) and HDL-cholesterol (Spearman correlation: Rho = −0.144, *p* = 0.213). Levels of circulating C3 in FH patients did not significantly vary in relation to the severity of total-plaque burden (Figure 1C). Circulating levels of C-reactive protein (CRP) in FH patients were below 1mg/L in all subjects (median [IQR] mg/L: 0.035[0.020–0.258]).

### 2.3. C3 Alternative System Components in Human Advanced Atherosclerotic Lesions

**Figure 2:**
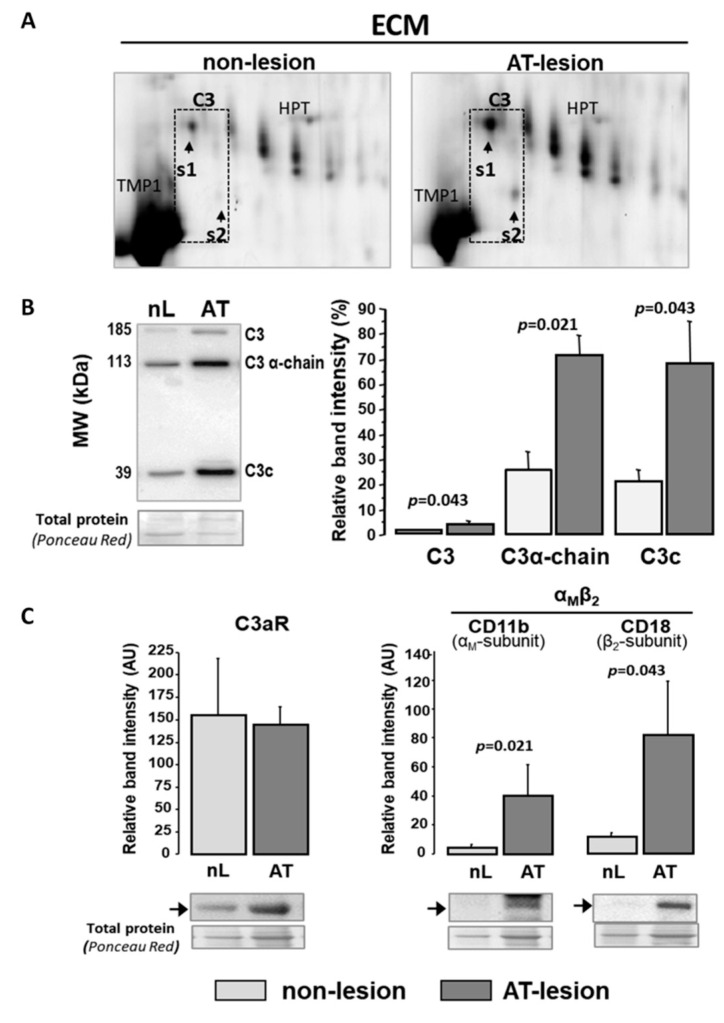
C3 complement components in human atherosclerotic lesions. (**A**) Representative 2D-gel images for protein extracts from normal and atherosclerotic ECM of human aortas (*n* = 3 independent with nL and AT segments). Arrow heads indicate position of spots identified as C3 by MALDI-ToF/ToF (Mascot score = 82). Tropomyosin-1 (TMP1) and Haptoglobin (HPT) are indicated as landmarks. (**B**,**C**) Western Blot analysis for C3 and C3 receptors in total extracts from non-lesion (nL) and atherosclerotic (AT) segments of human aortas (*n* = 4 independent arteries with nL and AT segments). (**B**) denotes C3 activation products, and **C** refers to receptors C3aR and α_M_β_2_ for C3a and iC3b/C3b (C3 activation products). Band relative intensity was normalized against total protein, visualized with Ponceau Red staining and expressed as mean ± SEM. The antibody against C3 recognizes complete full-length C3 (C3), C3 α-chain (C3 α-chain) and C3 α-chain-fragment 2 from C3c (C3c), product of degradation of iC3b. *p* < 0.05 was considered statistically significant (Mann–Whitney test).

Human atherosclerotic aortas were obtained from the Eulalia Study Biobank (ICCC) [28]. The extracellular matrix (ECM) of human aortas was enriched in active components of the C3 system with a significantly different proteomic signature in atherosclerotic areas when compared to lesion-free segments. Specifically, by two-dimensional electrophoresis (2DE) and MS/MS (MALDI-ToF/ToF), the complement-protein C3 was consistently identified as two independent spots (s1 and s2) in the ECM of atherosclerotic lesions (Figure 2A and Figure S1), whereas only weaker or non-consistent signals for s1 and s2 were identified in the ECM of aortic segments without macroscopic evidence of atherosclerotic lesions. When analyzed by Western blot (Figure 2B), protein extracts from aortic ECM showed three different C3-positive bands corresponding, based on their molecular size, to the full-length molecule (185 kDa), the C3 α-chain (113 kDa, obtained after proteolytic loss of a four-arginine peptide) and the final proteolytic product C3c-fragment (39.5 kDa, α-chain). ECM extracts from atherosclerotic (AT) segments had >3-fold higher intensity in the C3-positive bands than protein extracts from non-lesion (nL) segments (AT vs. nL: *p* < 0.05 for intensity differences for each protein band).

Moreover, C3-activated fragment receptors were consistently detected in the cell–protein fraction (SDS fraction) of aortic extracts, both from control and atherosclerotic areas (Figure 2C). Western blot analysis showed significantly higher levels of the α_M_ and β_2_ subunits (CD11b and CD18, respectively) of the integrin α_M_β_2_ (iC3b/C3b receptor) in atherosclerotic segments, whereas the expression level of the anaphylatoxin C3a receptor (C3aR) did not significantly differ between non-lesion and atherosclerotic aortic segments. (Figure 2C).

**Figure 3:**
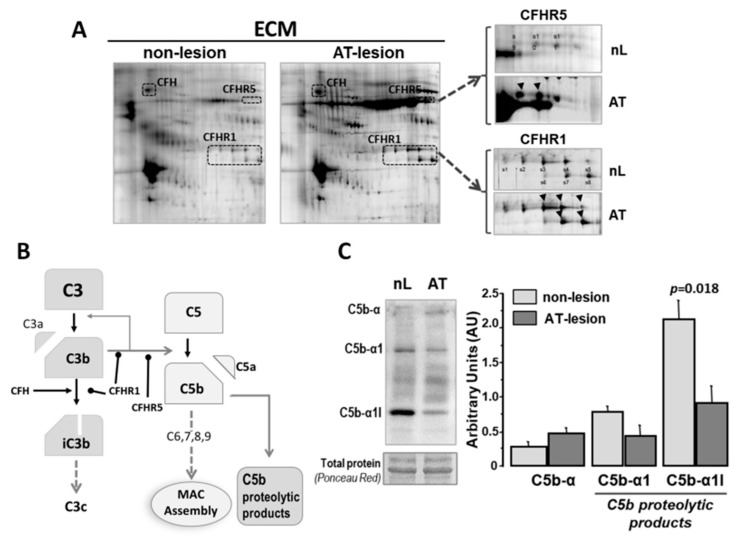
C3 complement regulatory components in human atherosclerotic lesions. (**A**) Representative 2D-gel images (*n* = 3 independent with nL- and AT segments) of the proteomic pattern corresponding to CFH, CFHR1 (s1 to s8) and CFHR5 (s9 to s11). Arrows indicate spots with significantly increased expression in AT-lesion samples (See Table 2). (**B**) Scheme of the proteolytic C3 cascade indicating steps regulated by CFHRs. (**C**) Western blot analysis of C5α chain proteolysis products in total extracts from non-lesion (nL) and atherosclerotic (AT) segments of human aortas (*n* = 4 independent arteries with nL and AT segments). Bars refer to arbitrary units of volume intensity in the Western blot bands (Mean ± SEM). Significance (*p* < 0.05, Mann–Whitney test) is indicated.

2DE-MS/MS analysis of human aortic ECM also evidenced the presence of C3 system regulatory components (Figure 3A), including the complement factor H (CFH) and CFH-related proteins CFHR1 and CFHR5. CFHR1 and CFHR5 were identified as two independent clusters of 7 and 4 spots (MW of 37–40 and 55 kDa), respectively. CFHR1 and CFHR5 clusters showed 2.5- and 3.8-fold (average of cluster spots) higher labeling signals in the ECM samples from atherosclerotic segments than those from normal aortic tissue (Table 2) and a different spot pattern distribution in normal and atherosclerotic segments of the aortic vessel wall (see Figure 3A). In addition, the complement component C5 (detected as α-chain) and its proteolytic products were consistently found in the aortic vessel wall, regardless of the presence of atherosclerotic lesions (Figure 3C). It is worth noting that the relative abundance of C5 fragmentation products (C5α1 and C5α1-I) was lower in atherosclerotic than in apparently normal segments.

### 2.4. C3 Alternative Pathway Components Expression in Vascular Wall Resident Cells

**Figure 4:**
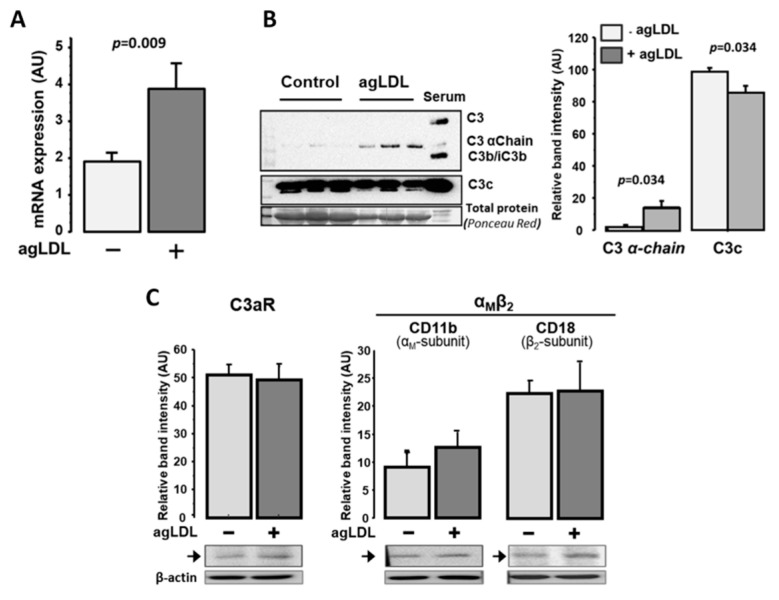
C3 alternative pathway components expression in human VSMCs. (**A**) mRNA quantification by real-time PCR using specific primers for human C3 in human VSMCs treated with or without agLDL (100 µg/mL). (**B**) Protein levels of C3 and C3-derived products in the supernatant of hVSMCs treated with or without agLDL (100 µg/mL). Human serum (Serum) was used as a positive control for C3 (*n* = 3 independent experiments). (**C**) Western blot analysis for C3a receptor (C3aR) and α_M_β_2_ (C11b/CD18) integrin (receptor for C3b/iC3b) in lysates of hVSMCs incubated with or without agLDL (100 µg/mL). Band intensity is given in arbitrary units as mean ± SEM and statistical significance (*p* < 0.05, Mann–Whitney test) is indicated (*n* = 4 independent experiments).

Complement factor C3 was consistently transcribed by human VSMCs (hVSMCs), and C3mRNA levels were up-regulated (1.7-fold, *p* < 0.05; Figure 4A) in lipid-loaded hVSMCs (24 h exposure to 100 µg/mL aggregated LDL agLDL). The significant protein expression of C3, receptor C3aR (anaphylatoxin receptor) and integrin α_M_β_2_ (CD11b/CD18) receptor for C3b/iC3b was also observed in hVSMCs (Figure 4B). In contrast, agLDL did not significantly affect the protein expression levels of the cell membrane receptors C3aR and integrin α_M_β_2_ (Figure 4C). Interestingly, the protein levels of C3 (C3 α-chain) were significantly increased in cell culture supernatants when hVSMCs were incubated in the presence of agLDL (Figure 4B). All together, these results indicate that there is a local synthesis of C3 components that were released to the ECM of atherosclerotic plaques.

### 2.5. Exogenous C3 Proteolytic Products, AgLDL and VSMC Function

**Figure 5:**
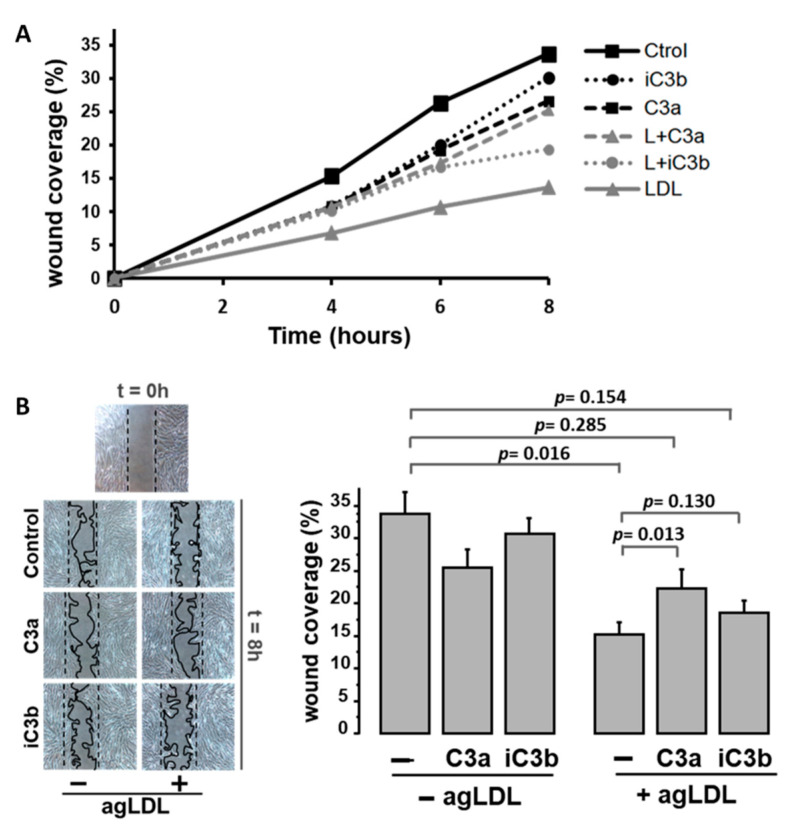
Effects of exogenous C3 proteolytic products on migration of human lipid-loaded hVSMCs. Results for wound coverage (%) in an in vitro model of wound repairing of FCS-stimulated hVSMCs treated with/without agLDL (100 µg/mL) in the presence or absence of C3a (10 nM) or iC3b (100 nM), (*n* = 6 independent experiments in duplicates). (**A**) Time-course for wound coverage by hVSMCs. *Ctrol and LDL*: cells incubated in the absence of C3 products, without or with agLDL, respectively. *iC3b and L+iC3b*: cells incubated with iC3b without or with agLDL. *C3a and L+C3a*: cells incubated with C3a without or with agLDL. (**B**) Representative microphotographs of wound-repairing model by hVSMCs taken at 0 and 8 h after inducing double-side injury. Bar diagrams refer to quantitative values for the % of wound covered area after 8 h injury. Band intensity is given in arbitrary units as mean ± SEM (*n* = 6 independent experiments in duplicates). *p* < 0.05 was considered statistically significant (Mann–Whitney and Kruskal–Wallis tests).

Lipid-loaded VSMCs have an impaired migration rate and cell attachment dynamics [29,30,31]. As shown in Figure 5, exogenously added C3 proteolytic products (10 nM C3a or 100 nM iC3b) partially reversed the impairment of the human VSMC (hVSMC) repair function induced by aggregated LDL (agLDL) to levels that did not differ significantly from the wound repairing capacity of hVSMC control cells. C3a induced a significant increase in the migrating capacity of lipid-loaded hVSMCs into the wound area. A similar trend, although non-significant, was observed with iC3b. C3 proteolytic products did not affect the wound-repairing capacity of control VSMCs (not exposed to agLDL).

**Figure 6:**
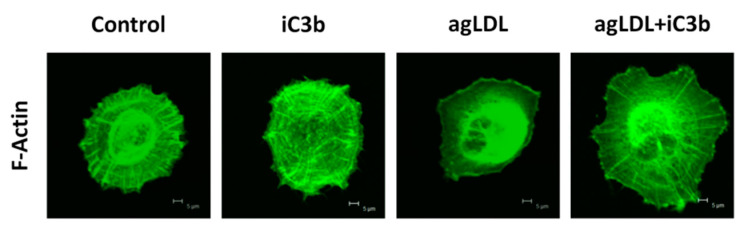
iC3b induces actin fiber polymerization and cytoskeleton rearrangement in hVSMCs exposed to agLDL. Confocal microscopy images of attached hVSMCs, 60 min after seeding. Representative photomicrographs of control cells and agLDL treated cells, in the presence/absence of exogenous iC3b (100 nM). Cells were labelled for F-actin with Alexa Cy3 488-phalloidin.

Moreover, the addition of iC3b (100nM) to hVSMCs in adhesion experiments induced a significant increase in the attachment capacity of the cells, both in the absence and presence of agLDL. The effect was more evident (higher percentage of increase) and more prolonged in time (up to 2 h after seeding) in the lipid-loaded hVSMCs compared to cells not exposed to agLDL (Table 3). In addition, exogenously added iC3b enhanced the organization of the F-actin cytoskeleton during cell adhesion. This was especially evident in hVSMCs exposed to agLDL that otherwise did not show any organized net of actin fibers (F-actin positive), 60 min after seeding (Figure 6).

## 3. Discussion

The C3 complement system is an important mediator of innate immunity responses and a key component of the complement system. Accumulating evidence indicates that high levels of C3 account for an increased risk of cardiovascular disease in humans [19,22,32] especially in patients with metabolic pathologies [33]. To date, however, most of the studies addressed to investigate the involvement of the complement system in the atherosclerotic process have focused on the C5b-derived terminal pathway of the complement system (reviewed in [14,15]).

Previous studies primarily employed immunohistochemistry to localize C3 components in the human atherosclerotic vessel wall [18,19]. Here, using mass spectrometry-based proteomics, we identified a differential protein signature of the alternative C3 complement system in the intima layer of atherosclerotic lesions compared to macroscopically apparent normal segments of human aortas. It is important to note that the sequential protein extraction method used in this study made it possible to separately investigate proteins located in the extracellular matrix (ECM) and those in the cellular fraction of the intima. Using this approach, we revealed an enrichment of the C3 complement proteolytic products and regulators of the C3 cascade, such as the factor H/CFHR family in the matrisome (subset of non-structural regulatory proteins) of the human atherosclerotic ECM.

In addition to being a structural support to provide cell anchorage, the components of the ECM interact with vascular resident and infiltrated cells, regulating their phenotype and function [34]. Thus, our results strongly suggest that the C3 complement components present in the atherosclerotic ECM are key players in the outside-in signaling that occurs in key vascular cells involved in atherosclerosis progression. In agreement, we and others [35,36] have consistently identified anaphylatoxin receptors in human atherosclerotic arteries. In addition, this study evidenced increased levels of α_M_β_2_ integrin in the cell fraction of atherosclerotic lesions, supporting the relevance of the iC3b/C3b-mediated signaling in the atherosclerotic process.

An unresolved question, with apparently controversial findings to date, refers to the origin of the vascular components of the alternative complement system since the C3 complement is mainly synthesized in the liver [37,38]. In the present study, we evidenced elevated levels of circulating C3 in clinically asymptomatic patients with genetic diagnosis of FH and subclinical atherosclerosis (assessed by CTA) when compared with plasma levels in young healthy subjects, suggesting a maintained activation of the innate immune response in FH, although all patients were long-term treated with LLT as per the guidelines [27]. Interestingly, these results extend our own previous findings reporting on higher levels of cMVs derived from inflammatory cells, specifically monocyte- and lymphocyte-derived cMVs, in FH patients under long-term LLT [6]. In this respect, monocyte/macrophage has been described to express complement proteins in response to several pro-atherogenic conditions, including high levels of cholesterol [39]. It is worth noting, however, that all FH patients in our study had circulating levels of CRP below 1mg/L, showing very low systemic inflammation which might be related to the fact that all patients were treated according to the guidelines with the highest LLT for more than one year, and they had a well-compensated lipid profile. Further studies might help to gain a better understanding of the association between immune cell activation and inflammatory markers in heterozygous FH patients and their relevance for disease progression.

Plasmatic C3 was positively correlated with levels of LDL-C and Apo-B in the whole population under study (healthy subjects and FH patients), whereas no correlation was found with other lipid components, such as triglycerides and HDL-C. In agreement, a previous study based on two-dimensional electrophoresis analysis of serum samples from four FH patients and four healthy subjects identified a protein spot as C3 in the serum of FH patients and reported a positive correlation between levels of the C3a product and total plasma cholesterol [23]. From our results in FH patients, we could not exclude that the higher content of C3 in the atherosclerotic ECM results from the LDL flux into the intimal arterial wall and the retention of ApoB-rich lipoprotein particles in the intimal ECM space by binding to proteoglycans, which in turn favors their modification to form aggregates (agLDL) [40,41]. However, in the present study, although we found a local C3 accumulation in the atherosclerotic ECM of human aortas, the levels of C3 in the systemic circulation do not seem to be a sensitive measure of the plaque burden severity measured by CTA imaging in FH patients with subclinical coronary atherosclerosis. This finding prompts us to hypothesize that vascular resident cells may represent a main source of C3 in human atherosclerotic plaques.

Supporting our view, experimental studies on hyperlipidemic Apolipoprotein-E knockout (ApoE-KO) mice fed a high-fat diet evidenced C3mRNA up-regulation in aortic tissue, even before atherosclerotic plaque formation [23]. Interestingly, clonally expanding SMC, recently linked to neointimal formation and atherosclerotic plaque pathogenesis [42,43], overexpressed C3 complement factor in *ApoE*-/- mice fed a high-fat Western diet [22]. Due to the post-mortem condition of the aortic samples, we could not analyze mRNA expression in the human atherosclerotic lesions in the present study, but we evidenced that cultured human VSMCs express C3mRNA and that C3 complement is consistently over-expressed when cells are exposed to atherogenic agLDL for 24 h periods. In agreement with this, supernatants of lipid-loaded cells were enriched in the C3α chain, a C3 form that was not present in the agLDL, but significantly increased in the atherosclerotic ECM of human aortas, supporting the relevance of VSMCs as a source of C3 complement active forms in the lesions. In contrast, the C3c fragments detected in the atherosclerotic intimal ECM seem mainly to have a systemic origin, entering the arterial intima through the LDL. Indeed, the serum showed a strong signal for C3c fragments, the final proteolysis product derived from C3 complement activation, when analyzed by Western blot, and a similar fragment was also found in purified agLDL (Figure S2). C3c has been described as a biomarker of heart failure [44], periodontitis [45] or amyotrophic lateral sclerosis [46], but its role and function needs to be further investigated.

As shown by Wang et al., C3 expressed by the clonal SMC induced proatherogenic effects, including the paracrine regulation of macrophage inflammation and autocrine-induced SMC proliferation [22]. Among the C3 proteolytic fragments resulting from the C3 cascade activation, C3a and iC3b/C3b are widely recognized as highly bioactive molecules directly involved in the regulation of cell phenotype and function [47]. In particular, C3a has been described to act as a chemoattractant for neural crest cells [48] and, through its receptor C3aR, to control leukocyte recruitment and endothelial activation in cerebral microvessel inflammation [49].

We previously described that agLDL, resembling the LDL retained and aggregated in the ECM of the intimal layer in areas with atherosclerosis [40,50,51], is internalized by human VSMCs, inducing changes in their phenotype and impairing cell functions, such as adhesion and migration, mainly mediated by effects on actin-cytoskeleton dynamics and organization [31,52,53]. In the present study, we demonstrate that the inhibitory effect of agLDL on VSMC migration is ameliorated by the presence of exogenous C3a to a level that did not significantly differ from the migration capacity of the control group (non-exposed to agLDL) in an in vitro wound healing assay. Interestingly, C3a did not show a similar enhancing effect of cell migration in the control hVSMCs. Similarly, we found that exogenously added- iC3b promoted attachment during the cell adhesion and reorganization of the F-actin cytoskeleton network. This effect was more evident and maintained in lipid-loaded cells that otherwise would not present any organized actin cytoskeleton shortly after seeding.

In summary, our results demonstrated for the first time the presence and differential abundance of active products of the C3 system in the ECM of human atherosclerotic lesions. In addition, we provided evidence of the capacity of C3-derived products, beyond their well-known role in inflammation and immunity, to modulate the migratory and repair function of VSMCs that is impaired by LDL. These results suggest the C3 complement pathway is a novel player in vascular remodeling and in the progression of advanced human atherosclerotic lesions.

## 4. Materials and Methods

### 4.1. Human Samples

#### 4.1.1. Subjects with Familial Hypercholesterolemia and Healthy Volunteers

The present study included 49 subjects with a genetic diagnosis of heterozygous familial hypercholesterolemia (FH) and thus lifelong exposure to high LDL plasma levels and high risk of premature atherosclerosis from the SAFEHEART cohort. A group of young healthy volunteers (non-FH subjects, *N* = 28) from the same cohort was used as reference group to establish the normal plasma range of C3 levels in a healthy population, at very low risk of presenting subclinical atherosclerosis. Demographic and clinical data of the FH patients and the healthy volunteers are provided in Table 1 and Table S1. Neither the FH nor the healthy group included pregnant subjects. Cases of sepsis or infections and with history of cancer or suspected clinical cardiovascular events were excluded. This part of the study was approved by the Local Ethics Committee for Clinical Investigation in the Fundación Jimenez Diaz (CEIC-FJD; Madrid, Spain) (protocol number: 01/09) and was conducted according to the Declaration of Helsinki (2013), and written informed consent was obtained from all participants [54].

Coronary atherosclerotic plaque characterization was performed by computed tomographic angiography (CTA), as previously described [27]. Coronary atherosclerotic-plaque burden was characterized and quantified using the SAPC (QAngio CT (Research Edition V2.1.16.1; Medis Specials, Leiden, The Netherlands) software. SAPC measurements were performed by a blinded operator, unaware of any clinical or biochemical data. [27].

#### 4.1.2. Aortas and Coronary Arteries

Abdominal aortic tissue was obtained from the Biobank of the Eulalia Study on out-of-hospital sudden death [28]. Autopsy was performed within 18 h after death (age 34–79 years old) following the established forensic protocol [55,56], and the study was approved by the Institutional Ethical Committee for Clinical Investigation (Hospital Santa Creu i Sant Pau; Barcelona, Spain). The samples were processed immediately. After the removal of connective tissue and adherent blood, the specimens were divided into grossly homogeneous parts. Aortic wall segments were classified by their macroscopic appearance according to the presence and severity of atherosclerotic lesions. In this study, we compared macroscopically normal-appearing areas with atherosclerotic plaques (raised white or yellow-white plaques) obtained from the same artery (*N* = 4 individual aortas). From all segments, the intima layer was dissected from the media, snap-frozen in liquid N_2_ and stored at −80 °C.

To confirm the validity of the macroscopic classification, representative samples of each type of segment were examined histologically. To this aim, segments from each aortic tissue were embedded with paraffin, and 5µM sections were stained with Massons’s trichromic to identify cellular areas (Figure S3). The images were captured with an Olympus microscope Vanox AHBT3 (Hamburg, Germany) coupled with a Sony 3CCD color video camera and processed using Visilog (Sony ESPAC, San Jose, CA, USA) software (version 4.1.5).

#### 4.1.3. VSMC Culture and LDL Preparation

Primary human VSMCs (Cell Application, Inc., San Diego, CA, USA) were cultured in M199 medium containing 20% FBS and used between passage four and seven, as previously described [30]. Unless otherwise indicated, experiments were performed in subconfluent monolayers after incubation without or with aggregated LDL (agLDL; 100 µg/mL) for 16 h.

Human LDLs (density 1.019–1.063 g/mL) were purified by ultracentrifugation from pooled sera of normocholesterolemic volunteers, and agLDLs were generated by vortexing LDL (1 mg/mL), according to the initial method described by Guyton et al. [57] and as previously performed in our group [58]. This method has been shown to produce similar LDL aggregation as LDL versican incubation [59].

LDL protein concentration was determined using the bicinchoninic acid (BCA)-method (ThermoFisher, Rockford, IL, USA) and LDL purity assessed by agarose gel electrophoresis (SAS-MX Lipo-kit, Helena Biosciences, Gateheads, UK). LDL preparations were tested to exclude the presence of endotoxin (Limulus amebocyte lysate test, BioWhittaker, Walkersville, MD, USA) and potential bacterial contamination that could derive in confounding results, and this proved to be negative in all cases. LDLs used in the experiments were less than 48 h old. LDL oxidation in all LDL preparations was excluded by assessing thiobarbituric-acid-reactive substance (TBARS) formation, according to Ohkawa et al. [60] with slight modifications [61].

### 4.2. Tissue Processing and Extraction of ECM Proteins

Aortic ECM proteins were extracted according to Didangelos et al. [62]. Briefly, 300 mg segments of aortic tissue (intima layer) were diced in 8–10 pieces (approximately 2 × 2 mm² size) and washed 5 times with PBS containing 25 mM EDTA. ECM-soluble proteins were obtained by incubating the aortic samples for 4 h at room temperature (RT) under mild agitation at 800 rpm, with 0.5 M NaCl, 10 mM Tris (pH 7.5), supplemented with 25 mM EDTA (10:1 buffer volume to tissue weight). Tissue pieces were left to drop. Then, the supernatant was collected, cleaned up with desalting Zeba-Spin columns (ThermoFisher, Rockford, IL, USA) and precipitated overnight with 5 volumes of chilled 100% acetone at −20 °C. Proteins were re-dissolved in deglycosilation buffer (NaCl 150 mM; sodium acetate 50 mM; EDTA 10 mM, supplemented with 0.05 units of HeparinaseII; Chondroitinase ABC and Endo-β-Galactosidase). Then, tissue samples were incubated with 0.08% SDS (10:1 buffer volume to tissue weight) and 25 mM EDTA for a further 4 h (RT, mild agitation at 800 rpm). SDS supernatant was collected. Thereafter, tissue pieces were incubated for 48 h in guanidine-HCl buffer (4 M guanidine-HCl, 50 mM sodium acetate, pH 5.8 supplemented with 25 mM EDTA). After removing the guanidine with 100% ethanol, samples were centrifuged at 16,000× *g* (10 min) and stored at −80 °C. All buffers contained protease inhibitors (1 tablet/50 mL, Complete-EDTA free Roche) and phosphatase inhibitors (1%). Reagents were obtained from Sigma-Aldrich ((Merck KGaA, Darmstadt, Germany). The purity of each fraction was confirmed by Western blot with specific antibodies against β-actin (ab8228, Abcam, 1/1000); ColI (ab6308, Abcam, 1/1000) and AEBP1 (#250461, Antibodies Online, 1/500; Aachen, Germany), that specifically partitioned in the NaCl-, SDS- and guanidin-HCl-fractions (Figure S4).

### 4.3. 2D Electrophoresis/Mass Spectrometry Analysis

Proteins (150 µg) were identified by matrix-assisted laser desorption/ionization time of flight (MALDI-ToF/ToF) mass spectrometry (Bruker Daltonics Autoflex III Smartbeam; Bruker Daltonik GmbH, Leipzig, Germany) after separation by two-dimensional electrophoresis (2-DE) [63]. Differential protein pattern analysis by 2DE was performed with arterial segments (apparently normal and atherosclerotic) obtained from 3 independent aortas. To guarantee the highest homogeneity and ensure better comparability, protein extracts from all arterial segments (*N* = 6) were run simultaneously in an Ettan-Dalt-6 Device (GE-Healthcare, Uppsala, Sweden), and analysis was performed in duplicate. Protein spots in the gels were labeled by fluorescence (Flamingo labeling, Bio-Rad), scanned (Typhoon, GE-Healthcare, Uppsala, Sweden) and analyzed for differences in the protein pattern between groups with PD-Quest 8.0.1 software (Bio-Rad, Hercules, CA, USA). Quantification of the protein spot volume was performed with the PD-Quest 8.0.1 software (Bio-Rad, Hercules, CA, USA) as previously described [29,63]. Briefly, the software created a single master that included all gels included in the analysis. A relative value that corresponds to the single spot volume compared to the total volume of the spots in each gel was assigned to each spot in the gels. Afterwards, this value was subjected to background extraction, and the final intensity value was then normalized by the local regression model (LOESS) method included in the software [29,63]. For protein identification, mass spectrometry (MS) spectra were submitted to a MASCOT (Matrix Science Ltd, London, UK) search on Swiss-Prot 57.15 database using the following parameters: taxonomy *Homo sapiens*, mass tolerance 50–100, up to 2 missed cleavages; carbamidomethyl (C) as global modification and oxidation (M) as variable modification. Identification was accepted with a score higher than 56 for peptide mass fingerprint and 20 for MS/MS.

### 4.4. Western Blot and ELISA Assays

Protein antigen levels in total lysates, obtained with RIPA buffer (50 mM Tris HCl pH 8.0, 150 mM NaCl, 0.5% triton X-100, 0.5% Sodium deoxyclodate, 0.1% SDS) as we previously described [30], and from ECM extracts (see above), were analyzed by Western blot, as described previously [52] using the following primary antibodies: C3 (ab200199, dilution 1/2000, Abcam, Cambridge, UK ); C5 (Abcam ab11876, dilution 1/500); C3aR (Abcam ab126250, dilution 1/1000); CD11b (Abcam ab133357, dilution 1/1000); CD18 (Abcam ab119830, dilution 1/500); Human β-actin (Abcam ab8226, dilution 1/5000) and total protein (Ponceau staining) were used as loading controls. Western blot bands were visualized by chemiluminescence using a peroxidase enzymatic reaction (Supersignal, ThermoFisher, Rockford, IL, USA)) and quantified with a ChemiDoc™ XRS system using Image Lab software (Bio-Rad, Hercules, CA, USA).

Quantitative plasma analysis of C3 was performed by a commercial double antibody sandwich enzyme-linked immunosorbent assay (AssayPro EC2101-1, St Charles MO, USA) with a lower limit of detection of 83 ng/mL calculated by 2SD from the mean of a zero standard. The intra-assay and inter-assay precision were CV < 5.2 and <8.9%, respectively.

### 4.5. RNA Extraction and Real-Time PCR Analysis

Total RNA was extracted from areas with migrating VSMCs (wound border) or non-migrating cells after 6 h of wounding (Figure S3) or from growth-arrested cells (maintained 18 h in M199 without FBS supplementation) using an RNeasy Mini Kit (Qiagen, ref. 74104), according to the manufacturer’s instructions. RNA concentration was determined with a NanoDrop ND-1000 spectrophotometer (NanoDrop Technologies), and purity was checked with the A260/A280 ratio.

mRNA levels were analyzed by real-time PCR [59] using an RT^2^ Profiler PCR targeted array for human cell motility (Qiagen; Cat. no. 330231 PAHS-128ZA) to compare gene expression profiles between migrating and non-migrating hVSMCs and with TaqMan fluorescent real-time PCR probes (ThermoFischer, Rockford, IL, USA)) to quantify C3 (Hs00163811-m1). Human GAPDH (4326317E) was used as an endogenous control. Samples were analyzed in duplicate, and only mRNAs with expression levels below 32 cycles were accepted.

### 4.6. Cell Adhesion and Wound-Healing Assays

Migration studies were performed with human VSMCs seeded in a culture insert (ibidi 2-well culture insert, ibidi GmbH, Martinsried, Germany) and left in M-199 with 10% FCS with or without 100 µg/mL agLDL until cell confluence was achieved. When indicated, C3a (10 nM) or iC3b (100 nM) were added to the culture medium 1 h before stimulation with agLDL and maintained during the assay. After removing the culture inserts, cells were washed with PBS and maintained in M199 migration medium (10% FCS) with its corresponding treatment for a total of 8 h. Migration on the cell-depleted area was controlled using an inverted microscope (Leica DMIRE2, Wetzlar, Germay) with a 10× lens magnification. Images were taken at 2 h intervals. During migration, cells were maintained at 37 °C in a humidified atmosphere of 5% CO_2_. The cell-free area of each field was quantitatively determined using ImageJ software. Changes in the viability of the cells due to agLDL had been previously excluded [64].

Cell attachment studies were performed as previously described [53]. Briefly, subconfluent cultures of VSMCs were incubated with or without iC3b (100 nM), in the presence/absence of agLDL (100 µg/mL) for 16 h. Cells were then harvested with trypsin, suspended in 5% FBS-containing medium and seeded (1 × 10^5^ cells) on FBS-coated glass bottom dishes in the presence or absence of iC3b (100 nM) and/or agLDL (100 µg/mL). At different time periods (30 min, 1 h and 3 h), attached cells were released by trypsination, stained with trypan blue for determination of cell viability and counted in a Neubauer chamber. Alternatively, at these time periods, cells were fixed with 4% paraformaldehyde for immunolabeling and confocal microscopy.

### 4.7. Confocal Focal Microscopy

Cells fixed with 4% paraformaldehyde were permeabilized (0.5% Tween-PBS), blocked with 1% bovine serum albumin (BSA), immunolabeled for F-actin and analyzed by confocal microscopy as previously described [30,31,52] using Alexa Fluor 633 or 488 phalloidin (Molecular Probes) on a Leica TCS SP2-AOBS inverted fluorescence microscope (Leica Microsystems Heidelberg GmbH, Mannheim, Germany). Fluorescent images were acquired in a scan format of 1024 × 1024 pixels at intervals of 0.1 mm (20 slides) and processed with the TCS-AOBS software (Leica). Maximal intensity projection values were calculated using the LASAF Leica Software and given as AU/mm^2^.

### 4.8. Statistical Analysis

Results are presented as the mean ± SEM (standard error of the mean), except when indicated. Outlier expressions were excluded by Chauvenet’s criterion. Sample distribution was verified by the Shapiro–Wilk test. Statistical differences between groups were analyzed by non-parametric tests (Kruskal–Wallis or Mann–Whitney), as indicated. StatView software (Abacus Concepts) and SPSS Statistics Version 21.0.0 (SPSS, Chicago, IL, USA) were used for statistical analysis, and a *p*-value < 0.05 was considered statistically significant.

## Figures and Tables

**Table 1:** Demographic, biochemical and clinical variables: Familial hypercholesterolemia and healthy subject groups.

	Familial Hyperchlesterolemia *n* = 49	Healthy Subjects *n* = 28
**Demographic Characteristics; mean ± SD**		
Female/male, n	18/31	16/12
Age, years	38.6 ± 11.3	24.5 ± 4.6
Risk Factors; n (%)		
Smokers	14 (29)	12 (43)
Hypertension	1 (2)	0 (0)
Diabetes mellitus	0 (0)	0 (0)
Dyslipidaemia	48 (98)	0 (0)
**Biochemical Data, Mean ± SD**		
Total cholesterol, mg/dL	282 ± 72	170 ± 20
Triglycerides, mg/dL	104 ± 67	77 ± 35
HDL cholesterol, mg/dL	47 ± 11	56 ± 15
LDL cholesterol, mg/dL	221 ± 78	99 ± 15
Apo AI, mg/dL	135 ± 20	139 ± 29
Apo B, mg/dL	134 ± 41	61 ± 10
Lipoprotein(a), mg/dL	42 ± 35	18 ± 21
Glucose, mg/dL	89 ± 9	78 ± 9
C-reactive protein	1.86 ± 2.6	0.73 ± 0.2
**Subclinical Atherosclerotic Disease; (%)**		
Plaque burden, %	23.5 ± 6.3	-
Calcium burden, %	2.2 ± 2.5	-
Non-calcium burden	21.3 ± 5.3	-
**BAckground Medication; n (%)**		
Angiotensin-converting-enzyme inhibitors	0 (0)	0 (0)
Angiotensin II receptor blockers	1 (2)	0 (0)
Beta-blockers	0 (0)	0 (0)
Diuretics	2 (4)	0 (0)
Statins *	39 (80)	0 (0)

SD: standard deviation * Includes: rosuvastatin, ezetimibe, atorvastatin, simvastatin, lovastatin, pravastatin, Fluvastatin, pitavastatin, resins, and fibrates. Healthy subject population was used to establish the C3 range in a healthy group.

**Table 2:** C3 complement-system proteins identified on advanced atherosclerotic-lesion human aortas.

Fraction	Protein	UniProt-Code	Gene-Code	MS-Score *	Seq/Int Cov. (%) *	MW (kDa)	pI-Value	Fold-Change
sb-ECM	*Complement Factor H*	P08603	*CFH*	102	9.9/78.4	143.7	6.20	≈(1.2)
Sb-ECM	*Complement Factor H-related protein 1*	Q03591	*CFHR1*	120	28.2/95.3	38.8	8.70	↑(2.4)
Sb-ECM	Complement Factor H-related protein 5 **	Q9BXR6	*CFHR5*	28	--	66.4	7.00	↑(3.9)
Lb-ECM	*Complement C3*	P01024	*C3*	83	9.4/76.7	188.6	6.00	↑(2.9)

Proteins were identified by peptide mass fingerprint and confirmed by MS/MS by MALDI ToF/ToF. * Mascot Score, sequence and intensity coverage are expressed as representative values. ** Only identified by MS/MS.

**Table 3:** Effect of iC3b on cell adhesion capacity in the absence and presence of agLDL.

	Control	+iC3b		
		30 min	60 min	120 min
−agLDL	100.0 ± 0.0	93.9 ± 4.4	115.9 ± 4.8 *	93.7 ± 14.0
+agLDL	100.0 ± 0.0	65.4 ± 1.8	122.0 ± 2.6	177.1 ± 42.3 *

Results refer to the number of attached cells from a total of 1 × 105 seeded cells, expressed as percentage of attached cells in the groups non-receiving iC3b (controls). Cell viability was in all cases >95% as determined by trypan blue staining. Results are given as mean ± SD of three independent experiments in duplicates. * *p*-values for comparison (Mann–Whitney test) between cells with/without agLDL −/+ addition of exogenous iC3b at 3 different time points. * *p* < 0.05.

## Data Availability

Not Applicable.

## Supplementary Materials

The following are available online at https://www.mdpi.com/article/10.3390/ijms22105122/s1.
