# Supplementary material for: Alternative C3 Complement System: Lipids and Atherosclerosis
Source: Int J Mol Sci. 2021 May 12;22(10):5122. doi: 10.3390/ijms22105122 (PMC8151937; doi:10.3390/ijms22105122)
Supplement: Supplementary file 1 [file ijms-22-05122-s001.zip › ijms-1206511-supplementary.pdf]

Supplementary Information

Alternative C3-complement system: lipids and atherosclerosis

Maisa Garcia-Arguinzonis[1], Elisa Diaz-Riera[1], Esther Peña[1,2] , Rafael Escate[1,2], Oriol Juan-Babot[1], Pedro Mata[3], Lina Badimon[1,2,4#], Teresa Padro[1,2#*]

# *Both authors contributed equally*

[1]Cardiovascular Program-ICCC, Research Institute- Hospital Santa Creu i Sant Pau, IIB-Sant Pau, Barcelona.Spain

[2]Centro de Investigación Biomédica en Red cardiovascular (CIBERCV) Instituto de Salud Carlos III, Madrid, Spain.

[3]Fundación Hipercolesterolemia Familiar, Madrid, Spain

[4]Cardiovascular Research Chair, UAB, Barcelona, Spain

Address for corresponding author (*):

Dr Teresa Padro
Cardiovascular Program-ICCC
Research Institute Hospital Santa Creu i Sant Pau
Sant Antoni Mª Claret 167, 08025 Barcelona, Spain
Phone: +34 935565886
Fax: +34 935565559
E-mail: tpadro@santpau.cat

Table S1: Demographic, biochemical and clinical variables: Familial hypercholesterolemia and healthy subject groups

	FH population n=49	Reference group n=28
Female/male, n	18/31	16/12
Age, years	44.69±10.5	24.5±4.6
BMI	25.7±3	22.4±2.9
**RISK FACTORS; n (%)**		
Active smoking	11 (22)	12 (43)
Hypertension	2 (4)	0 (0)
Type 2 diabetes	1 (2)	0 (0)
SAFEHEART risk 5 years, %	1.00±0.76	-
SAFEHEART risk 10 years, %	2.13±1.6	-
**BIOCHEMICAL DATA; MEAN ± SD**		
Total cholesterol, mg/dL	206.7±38	169.7±21
Triglycerides, mg/dL	90.6±49	76.8±35
HDL cholesterol, mg/dL	52.1±13	55.7±16
Non-HDL cholesterol, mg/dL	154.6±40	114.0±16.7
LDL cholesterol, mg/dL	136.3±36	98.6±15
Apo AI, mg/dL	126.5±17	139.5±29
Apo B, mg/dL	106.6±24	61.5±10
Lipoprotein a, mg/dL	48.8±46	18.5±21
Glucose, mg/dL	91.0±9	78.3±9
**SUBCLINICAL ATHEROSCLEROTIC DISEASE; (%)**		
Plaque burden, %	23.5±6.3	-
Calcium burden, %	2.2±2.5	-
Non-calcium burden, %	21.3±5.3	-
**BACKGROUND MEDICATION; n (%)**		
Angiotensin-converting-enzyme inhibitors	0 (0)	0 (0)
Angiotensin II receptor blockers	1 (2)	0 (0)
Beta-blockers	0 (0)	0 (0)
Diuretics	2 (4)	0 (0)
Statins*	49 (100)	0 (0)
Lipid-lowering treatment, years	14.8±6.7	

SD: *standard deviation. *Includes: rosuvastatin, ezetimibe, atorvastatin, simvastatin, lovastatin, pravastatin, fluvastatin, pitavastatin, resins, and fibrates.*

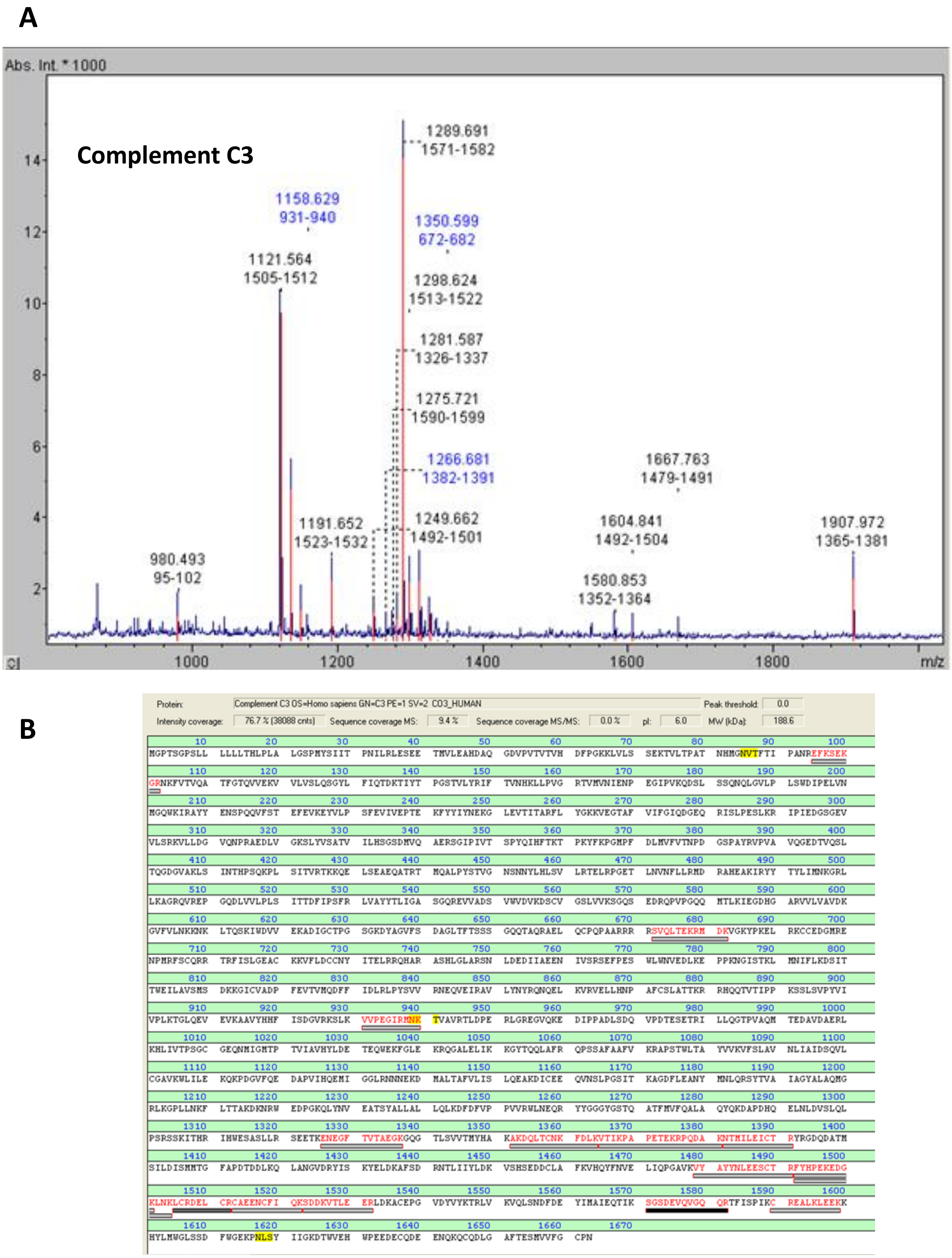

Figure S1: **Mass Spectrometry Analysis.** Identification by MALDI ToF/ToF of Complement C3 on 2DE-PAGE from soluble protein fraction of ECM from human aortas, identified by a MASCOT search on Swiss-Prot 57.15 database with a Score of 82, a sequence coverage of 9.4% and intensity coverage of 76.7%. To identify the following parameters were used: taxonomy *Homo sapiens*, mass tolerance 50–100, up to 2 miss cleavage; global modification: carbamidomethyl (C); variable modification: oxidation (M). Identification was accepted with a score higher than 56. Spectrum(A) and sequence Coverage (B) obtained with a Smartbeam AutoFlex III and visualized with BioTools Software (version 3.02, Bruker)

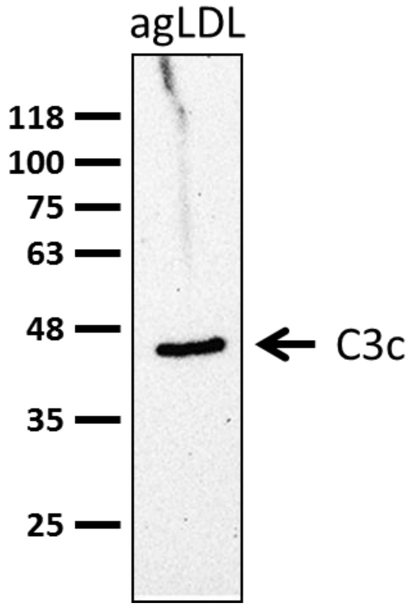

Figure S2: Western Blot analysis for C3 in agLDL. 250µl of agLDL (1ug/mL) was precipitated with ice cold acetone O.N. and resuspended in 25µl of 50mM Tris-HCl pH 8.0 and analyzed by western blot using C3 primary antibody (Abcam ab200199, dilution 1/2000) and visualized by chemiluminescence using a peroxidase enzymatic reaction (Supersignal, Pierce) and quantified with a ChemiDoc™ XRS system using using Image Lab software (Bio-Rad).

HUMAN AORTAS

no Lesion Area (nL) | **Atherosclerotic Lesion (AT)**

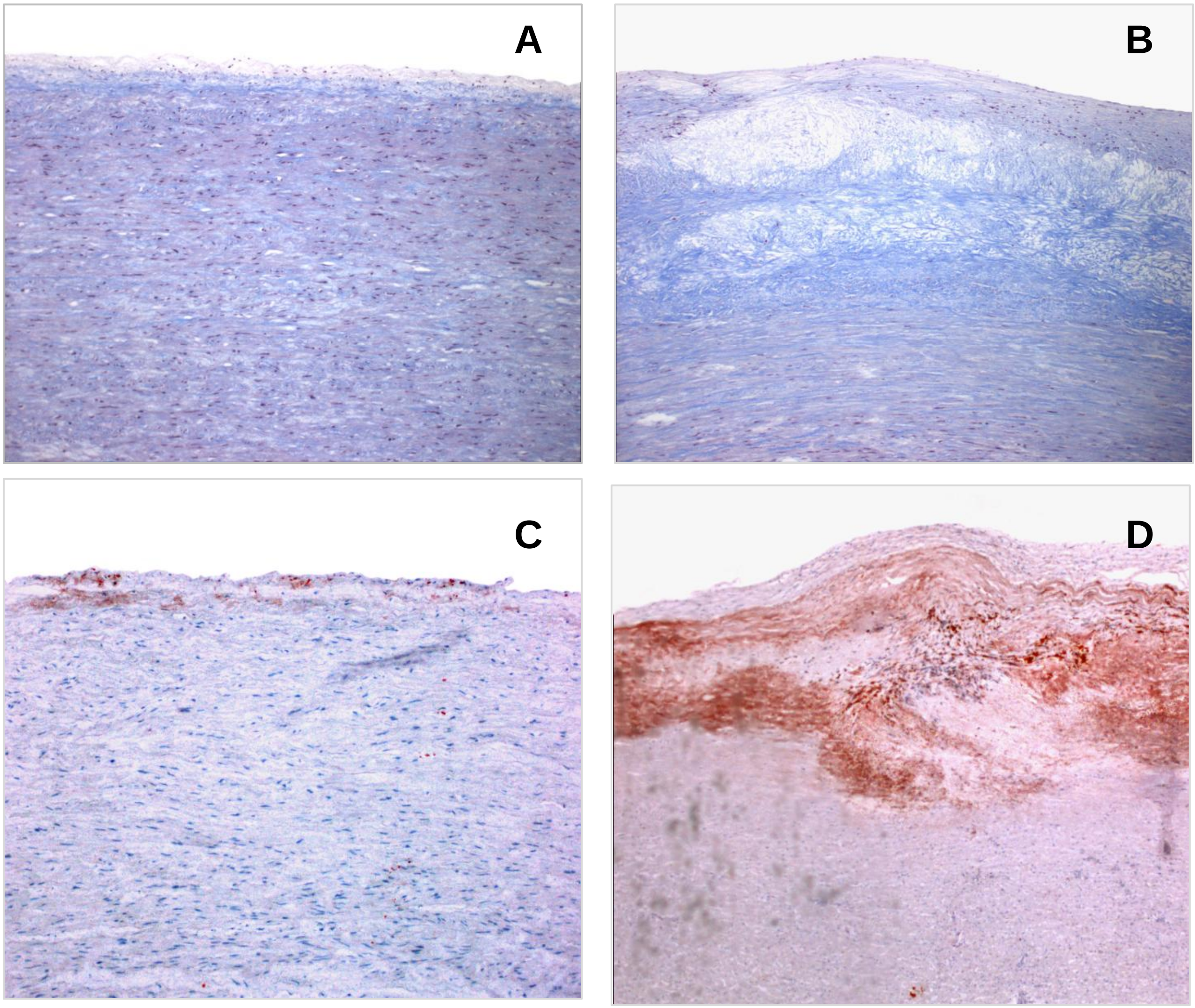

Figure S3: Histological analysis of human aorta. **A** and **C** Macroscopically normal-appearing areas (nL: no lesion areas) or **B** and **D** Areas with atherosclerotic plaques (AT segment). Aortic segments were embedded with paraffin and 5µM sections were stained with Massons's trichromic to identify cellular areas. **C** and **D** Representative of atherosclerotic plaque segment stained for lipids with Oil Red O. The images were captured with an Olympus microscope Vanox AHBT3 coupled with a Sony 3CCD color video camera and processed using Visilog (Sony ESPAC) software (version 4.1.5). Magnification x60

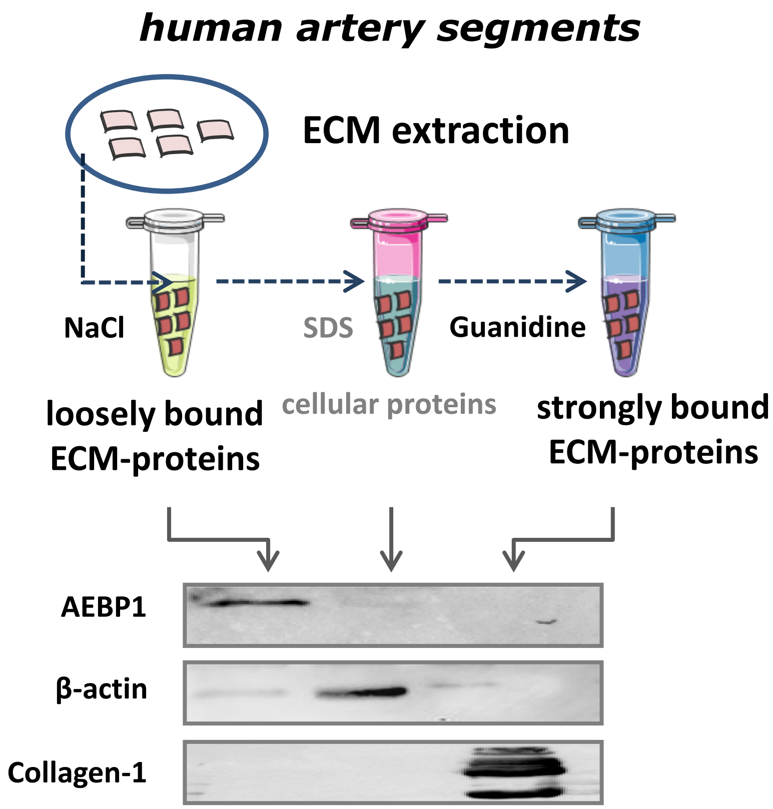

Figure S4: Schematic diagram representing tissue extraction of human aorta. Segments of human aorta with or with advanced atherosclerotic lesions and without atherosclerosis obtained from sudden death cases were sequentially extracted to obtain the ECM protein fraction as described in the Material and Methods section. Extraction purity was confirmed by western blot using specific antibodies for each fraction: AEBP1 (Adipocyte enhancer-binding protein 1) for the loosely bound ECM protein fraction; β-actin for the intracellular proteins fraction and Collagen-1 the for strongly bound ECM-protein fraction.
